# Nanoscale Evaluation of the Degradation Stability of Black Phosphorus Nanosheets Functionalized with PEG and Glutathione-Stabilized Doxorubicin Drug-Loaded Gold Nanoparticles in Real Functionalized System

**DOI:** 10.3390/molecules29081746

**Published:** 2024-04-12

**Authors:** Thisari Maleesha Gunathilaka, Masaru Shimomura

**Affiliations:** Graduate School of Science and Technology, Shizuoka University, 3-5-1 Johoku, Chuo-ku, Hamamatsu 432-8011, Shizuoka, Japan; u.m.thisari.m.g.20@shizuoka.ac.jp

**Keywords:** black phosphorus, degradation behavior, degradation stability, nanoscale evaluation, PEG and gold functionalization

## Abstract

Two-dimensional black phosphorus (2D BP) has attracted significant research interest in the field of biomedical applications due to its unique characteristics, including high biocompatibility, impressive drug-loading efficiency, phototherapeutic ability, and minimal side effects. However, its puckered honeycomb lattice structure with lone-pair electrons of BP leads to higher sensitivity and chemical reactivity towards H_2_O and O_2_ molecules, resulting in the degradation of the structure with physical and chemical changes. In our study, we synthesize polyethylene glycol (PEG) and glutathione-stabilized doxorubicin drug-assembled Au nanoparticle (Au-GSH-DOX)-functionalized BP nanosheets (BP-PEG@Au-GSH-DOX) with improved degradation stability, biocompatibility, and tumor-targeting ability. Transmission electron microscopy, X-ray photoelectron spectroscopy, and Raman spectroscopy indicate the nanoscale degradation behavior of synthesized nanoconjugates in three different environmental exposure conditions, and the results demonstrate the remarkable nanoscale stability of BP-PEG@Au-GSH-DOX against the degradation of BP, which provides significant interest in employing 2D BP-based nanotherapeutic agents for tumor-targeted cancer phototherapy.

## 1. Introduction

Black phosphorus (BP), a two-dimensional (2D) material, has attracted significant research interest surpassing other 2D materials such as graphene, MXenes, and transition-metal dichalcogenides, owing to its outstanding chemical, optical, mechanical, electrical, and thermal properties [1,2,3,4]. BP consists of stacked monolayers (phosphorene) to form layered crystalline material by van der Waals interactions, owing to direct and tunable band gaps ranging from 0.3 eV for bulk BP to 2.0 eV for phosphorene [5,6,7]. Consequently, BP has gained considerable attention for various applications, mainly in the field of biomedicine, as a potential candidate for cancer therapeutics, owing to its promising optical, physicochemical, and electronic properties [8,9,10,11]. In addition, BP is an ideal photothermal agent, owing to its high near-infrared extinction coefficient and excellent photothermal conversion efficiency, making BP an effective choice for photothermal therapy applications [12,13].

BP can incorporate drug molecules into effective drug delivery systems, owing to its extensive surface area [14,15]. One of the most commonly used chemotherapeutic drugs is doxorubicin, which is effective against various types of cancer by inhibiting the topoisomerase II enzyme in tumor cells, resulting in DNA damage and ultimately cell death [16,17]. The addition of inorganic compounds, such as gold nanoparticles (Au NPs) and BP nanosheets (NSs), can improve the capacity of delivering anticancer drugs. Additionally, Au NPs are effective in enhancing photothermal activity by increasing light absorption, generating more heat and facilitating heat dissipation through the functionalized BP system [18,19,20,21,22]. Despite the considerable potential of BP in a wide range of applications, it presents a significant challenge due to its ultimate degradation in the presence of water and oxygen, resulting in structural and compositional changes. This is attributed to its honeycomb lattice structure with lone-pair electrons. However, BP undergoes nontoxic degradation to produce phosphates and phosphonates, which are well tolerated in the human body [23,24,25,26]. Thus, various strategies have been implemented to enhance the stability of BP, including functionalization, passivation via solvents, the incorporation of polymers, and the application of capping layers [27,28,29,30,31].

Covalent and noncovalent functionalization strategies are mainly employed to mitigate the degradation of BP. Covalent functionalization involves chemically bonding molecules to reactive lone pairs on the BP surface, which prevents decomposition [32]. Noncovalent functionalization, on the other hand, involves passivating BP nanoflakes through noncovalent interactions, thereby impeding their degradation [27]. In addition, solvent passivation can provide BP with protection against oxidizing agents, while polymer coatings improve resistance to oxidation by maintaining its significant properties [27,33]. Although it is well documented that the ambient degradation of the BP flakes of functionalization with different surface-modifying agents, such as poly lactic-co-glycolic acid (PLGA) [26], polydopamine (PDA) [34], poly(vinyl alcohol (PVA) [35], poly(methyl methacrylate) (PMMA) [36], Au, Te, Cu, Zr, and Ni and their metal ions [37,38,39,40], Al_2_O_3_, SiO_2_, MoS_2_, and graphene-based strategies [41,42,43,44], there is still a lack of knowledge on the nanoscale evaluation of BP oxidation. Consequently, no research has been conducted on BP degradation under different exposure conditions, such as inert conditions and lower temperatures with the dual functionalization of BP with PEG and Au, and only one study has attempted to investigate Au decoration concerning the effect of BP stabilization under ambient conditions, where the synthesis process differs from our study [37].

Therefore, the present study is the first to explore the chemical and morphological properties of glutathione (GSH)-protected doxorubicin (DOX) drug-loaded Au NPs on PEG-modified ultrathin BP NSs (BP-PEG@Au-GSH-DOX) at three different exposure conditions at inert conditions, lower temperature conditions, and ambient atmosphere via inspiring basic theory and research studies. This work expands upon a prior investigation conducted by our research group, which focused on the chemical and morphological features of BP-PEG@Au-GSH-DOX [45]. Additionally, we assessed the improved oxidation stability of BP-PEG@Au-GSH-DOX in relation to BP NSs degradation, a critical aspect in the development of BP NSs-based tumor-targeting cancer therapeutic systems. Transmission electron microscopy (TEM), X-ray photoelectron spectroscopy (XPS), and Raman spectroscopy were used to characterize and evaluate the stability of BP-PEG@Au-GSH-DOX against BP oxidation.

## 2. Results and Discussion

### 2.1. Morphology and Characterization of Synthesized Nanomaterials

**Figure 1:**
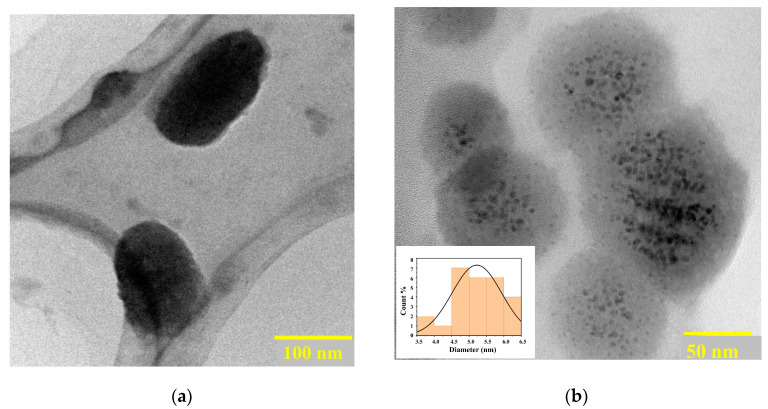
Transmission electron microscopic (TEM) images of (**a**) exfoliated BP NSs and (**b**) polyethylene glycol (PEG)-modified black phosphorous (BP) nanosheets (NSs) with glutathione-stabilized (GSH) doxorubicin (DOX) drug-incorporated Au nanoparticles (BP-PEG@Au-GSH-DOX NSs) and size distribution of Au NPs.

The liquid exfoliation technique was employed to obtain BP NSs from bulk BP crystals by ultrasonication in an ice bath. Selecting an exfoliation solvent plays a vital role in the stability of the exfoliated nanosheets. Thus, N-methyl pyrrolidone (NMP) was found to be the ideal solvent for BP NSs exfoliation due to its high surface tension and dielectric constant which was responsible for enhancing the stability of BP NSs and reducing the exfoliation time [46,47]. Exfoliated BP NSs were functionalized with PEG-NH_2_ and Au-GSH-DOX NPs to enhance the physiological stability, biocompatibility, tumor-targeting ability, and photothermal activity of the BP-PEG@Au-GSH-DOX nanoconjugate [18,48,49]. The TEM analysis (Figure 1a,b) shows the morphological structure of the exfoliated BP nanosheets (NSs) and BP-PEG@Au-GSH-DOX, with an average size of 100 nm approximately. Successful Au NP decorations with a uniform distribution (average size of ~10 nm) on the BP surface (Figure 1b) provide insight into the feasibility of the enhanced permeability and retention effect (EPR) of the synthesized BP-PEG@Au-GSH-DOX NSs for use as nanomedicine for tumor-targeted cancer therapies [14,17,50].

**Figure 2:**
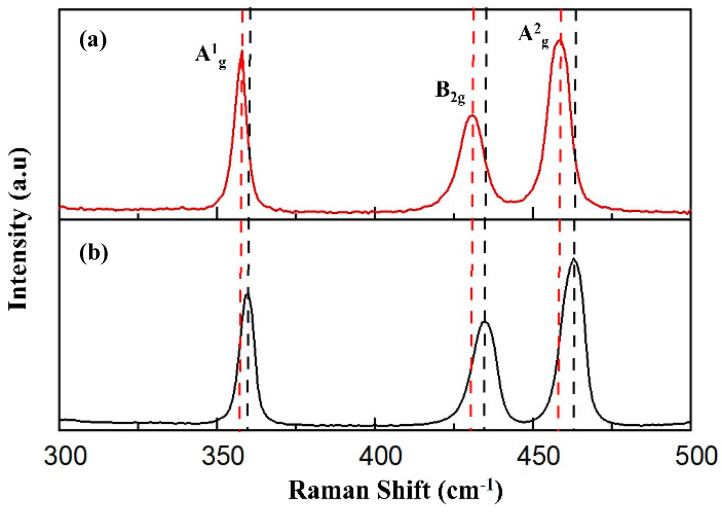
Raman spectra. (**a**) Polyethylene glycol (PEG) modified black phosphorous (BP) nanosheets (NSs) with glutathione-stabilized (GSH) doxorubicin (DOX) drug-incorporated Au nanoparticles (BP-PEG@Au-GSH-DOX NSs). (**b**) Exfoliated BP NSs.

Raman spectroscopy was conducted to study the chemical stability and structural modifications of Exf. BP and BP-PEG@Au-GSH-DOX. Two samples exhibit three prominent Raman peaks for the A^1^_g_ out-of-plane phonon mode and both the B_2g_ and A^2^_g_ in-plane phonon modes in Exf. BP (Figure 2a) and BP-PEG@Au-GSH-DOX (Figure 2b), confirming that the orthorhombic crystalline structure of BP was preserved during the synthesis of BP-PEG@Au-GSH-DOX without undergoing any structural transformations [51,52,53]. The three phonon modes (A^1^_g_, B_2g_, and A^2^_g_) of the BP-PEG@Au-GSH-DOX spectrum are redshifted due to the oscillation hindrance of phosphorus atoms by interacting with PEG and Au, thus reducing the corresponding Raman scattering energy. This observation further confirms the successful functionalization of BP to synthesize the BP-PEG@Au-GSH-DOX nanoconjugate [54,55,56].

### 2.2. Stability Evaluation of Exf. BP and BP-PEG@Au-GSH-DOX NSs

The chemical compositions and the relative percentage of the peak area of the oxidized phosphorus content of the Exf. BP NSs and BP-PEG@Au-GSH-DOX NSs were determined over time in three different environments [I. inert condition in an Ar glove box (23 ± 2 °C, 5 ± 2% RH), II. cooling temperature condition (4 ± 2 °C, 60 ± 5% RH), and III. ambient atmospheric condition (23 ± 2 °C, 71 ± 3% RH)] by using XPS analysis.

**Figure 3:**
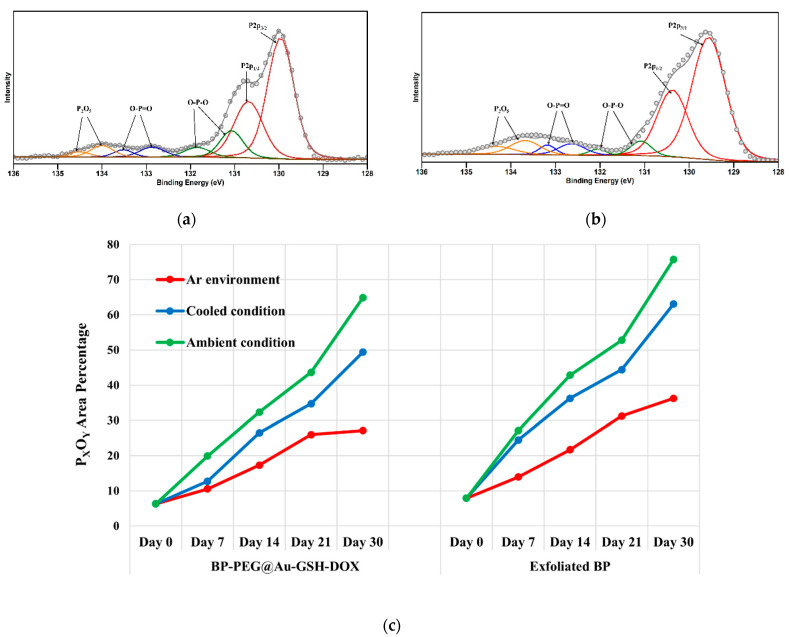
(**a**) P 2p X-ray photoelectron spectra of polyethylene glycol-modified BP NSs with glutathione-stabilized doxorubicin drug-incorporated Au nanoparticles (BP-PEG@Au-GSH-DOX NSs), (**b**) exfoliated black phosphorous nanosheets (BP NSs), (**c**) P_x_O_y_ shoulder peak of the XPS spectrum changes over a one-month period of polyethylene glycol (PEG)-modified black phosphorous (BP) nanosheets (NSs) with glutathione-stabilized (GSH) doxorubicin (DOX) drug-incorporated Au nanoparticles (BP-PEG@Au-GSH-DOX NSs) and exfoliated BP NSs.

Figure 3a,b depict the P 2p core levels of synthesized BP-PEG@Au-GSH-DOX and Exf. BP, respectively. Over time, both BP-PEG@Au-GSH-DOX and Exf. BP samples exhibited increased oxide shoulders in their XPS spectra, accompanied by a corresponding peak shift towards higher binding energies across three distinct exposure conditions. It is suggested that oxygen, due to its electronegative nature, draws electrons away from phosphorus, resulting in the formation of phosphorus oxide species with higher binding energies than those of the unoxidized P peaks. This leads to the production of P_x_O_y_ peaks between 131 and 136 eV which correspond to the +2, +3, and +5, P oxidation status (P → O-P-O/O-P=O → P_2_O_5_). These oxides then rapidly react with water in sample solutions to form dimethyl phosphate, phosphonic acid (H_3_PO_3_), and phosphoric acid (H_3_PO_4_) [26,57].

It is reported that there are specific degradation behaviors of BP, depending on the exposure of different oxidants such as H_2_O and O_2_ molecules. Specifically, O_2_ molecules initiate the oxidation of the basal surface of BP NSs, while H_2_O molecules promote defect-mediated oxidation [58]. The availability of both H_2_O and O_2_ facilitates the rapid degradation of nanosheets that contain a significant amount of high oxidation states of phosphorus (P^+3^ and P^+5^) [33]. Moreover, as the exposure time increased, the amount of oxidized phosphorus percentage also increased proportionately, which resulted in a higher oxidized P peak. This is indicated by the shift in the peak’s maximum binding energies, suggesting compositional alterations of phosphorus oxide composition [33,57]. By comparing the exposure conditions for phosphorus degradation in Exf. BP and BP-PEG@Au-GSH-DOX in water, Figure 3c exhibits the highest stability under an inert atmosphere in an Ar glove box with the comparatively lowest percentage of P_x_O_y_ area. This degradability is attributed to the reaction of BP with H_2_O molecules present in the sample solutions in oxygen-absent conditions [28,58]. The second highest stability is identified at cool temperature conditions and the lowest stability under ambient environmental exposure conditions in both Exf. BP and BP-PEG@Au-GSH-DOX samples. These observations are responsible for the fluctuations in oxygen and water molecules, resulting in substantial oxidation reactions with BP under various exposure conditions [57]. As per prior research, it has been established that solvent passivation occurs during degradation, which results in the formation of a protective layer against oxidants during the liquid exfoliation process. In our study, the use of NMP may have led to solvent passivation during the liquid exfoliation process, thereby increasing the stability of BP NSs during synthesis [58,59].

In contrast, the BP-PEG@Au-GSH-DOX sample shows remarkable resistance to degradation under three distinct conditions over a period of four weeks, suggesting greater stability against the oxidation of BP. The stability of these findings can be attributed to the formation of a protective layer on the surface of BP by PEG and Au NPs, which occupies energetic defect sites and reactive lone pairs and prevents oxidation [22,54,60,61]. This addresses the drawback of BP, which typically indicates higher degradation and limits its applications in drug synthesis. These interesting findings demonstrate the potential to enhance the stability of BP via effective functionalization for use in nano biotherapeutic applications.

In order to investigate the oxidation of BP in response to light irradiation, exfoliated BP-PEG@Au-GSH-DOX samples were kept in covered and uncovered borosilicate vials to regulate light exposure, and their degradability was evaluated under three different environmental conditions (inert conditions in an Ar glove box, cooler temperature, and ambient atmospheric conditions) over 14 days without employing any extra light source. XPS analysis was conducted three times (days 1, 7, and 14), and the percentage of phosphorus oxides was determined. Remarkably, similar observations were made for the samples inside both covered and uncovered containers, which may be attributed to the relationship between the layer number of BP nanoflakes and the light-induced oxidation [28,33,58]. Additionally, the observations were made on the degradation stability in the order of Ar environment condition ˃ cool temperature conditions ˃ ambient environment condition. However, further studies are required to verify this observation.

**Figure 4:**
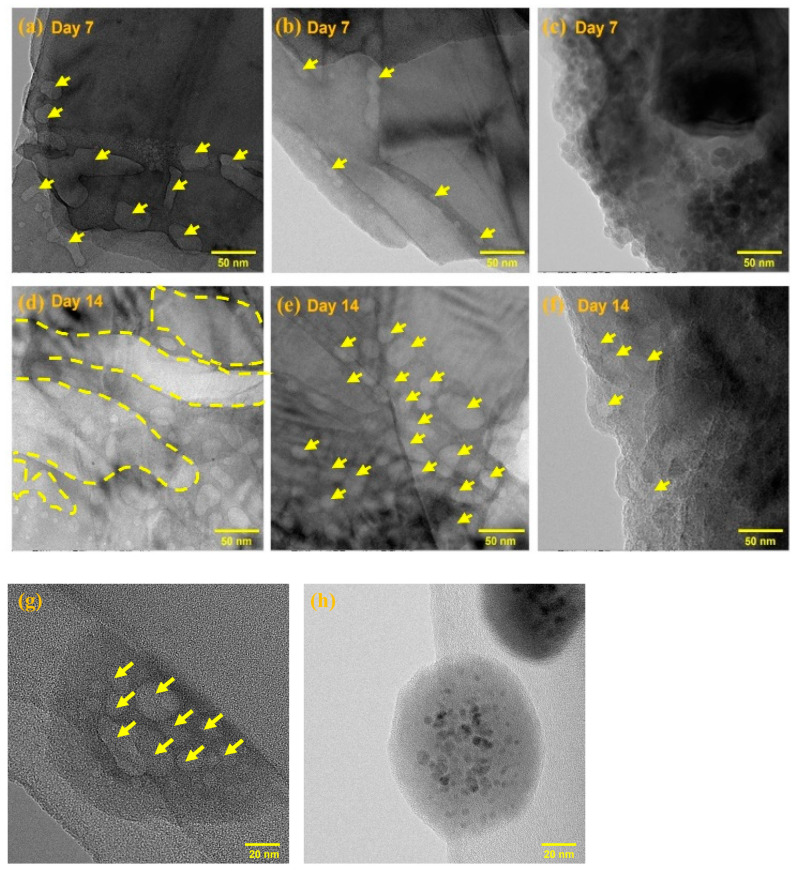
TEM analysis of (**a**,**d**) exfoliated BP, (**b**,**e**) PEGylated BP, and (**c**,**f**) BP-PEG@Au-GSH-DOX after 7 days and 14 days air exposure and (**g**) exfoliated BP and (**h**) BP-PEG@Au-GSH-DOX after 14 days in Ar glove box (yellow-colored arrows indicate the pitting areas on BP surface due to degradation).

The morphology of BP degradation in both Ar-filled glovebox and ambient exposure conditions was analyzed through TEM analysis using samples of Exf. BP, PEGylated BP, and BP-PEG@Au-GSH-DOX over 14 days. Figure 4a–f shows a significant difference in the surface morphology of Exf. BP, PEGylated BP, and BP-PEG@Au-GSH-DOX when exposed to ambient environmental conditions (23 ± 2 °C, 71 ± 3% RH). Similar to previous studies, Exf. BP exhibited pitting on its surface, particularly along the edges, which is caused by the degradation of surface oxidation due to oxygen and water exposure [57,62]. As the exposure time increased, significant pitting areas were observed in the 2D flakes of BP, with severe degradation. In contrast, the PEGylated BP NSs images in Figure 4b,e show fewer pitting areas at the edges during ambient exposure for one week. However, over time, significant degradation became visible with increasing pit diameters and wider pitting areas, ultimately causing damage to the surface structure of the BP NSs. In contrast, BP-PEG@Au-GSH-DOX exhibited remarkable stability and maintained its integrity on the surface of the BP for one week without any visible changes. Continuous ambient exposure caused only minor pitting at the edges, as indicated by the arrows, which confirmed the superior oxidation stability of the synthesized nanoconjugate under ambient conditions. Figure 4g,h compare the degradation behavior of Exf. BP and BP-PEG@Au-GSH-DOX under Ar environmental conditions. Exf. BP displays high surface damaging areas (indicated by arrows), whereas there are no significant noticeable damages in BP-PEG@Au-GSH-DOX, which further confirms the improved stability of the BP-PEG@Au-GSH-DOX.

**Figure 5:**
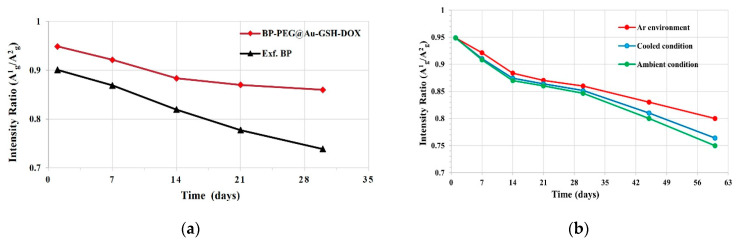
(**a**) Comparison of A^1^_g_/A^2^_g_ intensity ratio changes over time in polyethylene glycol (PEG)-modified black phosphorous (BP) nanosheets (NSs) with glutathione-stabilized (GSH) doxorubicin (DOX) drug-incorporated Au nanoparticles (BP-PEG@Au-GSH-DOX NSs) and exfoliated BP NSs and (**b**) BP-PEG@Au-GSH-DOX NSs in three environmental exposure conditions.

Raman spectroscopy offers valuable insights into sample degradation by evaluating the sensitivity of the A^1^_g_/A^2^_g_ intensity ratio of synthesized samples [33,37]. Initially, the intensity ratio changes are evaluated in the phonon modes of Raman spectrums of Exf. BP NSs and BP-PEG@Au-GSH-DOX-containing samples. As illustrated in Figure 5a, both samples exhibit a decrease in intensity over a period of one month. Notably, the A^1^_g_/A^2^_g_ intensity reduction in Exf. BP NSs is substantially greater than that of BP-PEG@Au-GSH-DOX, which further validated the superior stability of the successfully functionalized nanomaterial. To assess the changes in the A^1^_g_/A^2^_g_ intensity ratio in three distinct environmental conditions, BP-PEG@Au-GSH-DOX samples were exposed to inert conditions in an Ar glove box, reduced temperature, and ambient atmospheric conditions for four weeks, and we analyzed the A^1^_g_/A^2^_g_ ratio by subtracting the baseline (A^1^_g_/A^2^_g_ < 0.6 indicates the degradation of the BP surface). This evaluation method for BP degradation by oxidation has been supported by previous studies [26,63]. As depicted in Figure 5b, the samples in all three exposures display unoxidized basal planes, confirming the structural integrity of the BP NSs in BP-PEG@Au-GSH-DOX. This is achieved by occupying energetic defect sites in BP with PEG and Au NPs, thereby preventing the reaction between P and oxygen or water [64,65].

Evaluating the compatibility of BP-based nanotherapeutics with biological systems and determining their physiological stability is crucial for their potential use in biomedical applications. Bare BP NSs face limitations in physiological media containing salts, owing to the electron-screening effect, which can lead to instability through aggregation and precipitation [66]. Physical and chemical modifications of BP have been found to effectively enhance the stability of BP-based nanomaterials under physiological conditions, making them valuable therapeutic agents for clinical applications [8,9,64]. Recent studies reported that functionalizing BP NSs with PEG-NH_2_ through electrostatic interactions forms a protective layer on the surface of BP to shield it from possible oxidizing agents, resulting in enhanced stability in physiological media. For instance, Tao et al. confirmed the excellent stability of BP-PEG over one week in PBS and fetal bovine serum (FBS) media without significant changes in size and morphology [67]. Sun et al. and Wan et al. further confirmed the increased physiological stability of BP quantum dots after PEGylation, with excellent biocompatibility in both PBS and cell culture media, suggesting that the PEGylation of BP NSs is responsible for significant biocompatibility and physiological stability [68,69]. Moreover, the use of AuNPs creates a synergistic approach to enhance the stability of the BP nanostructures. This is achieved through mechanisms such as surface passivation, which forms a barrier to prevent direct contact with degradation, accelerating environmental factors, and charge transfer that modifies the electronic structure of BP, which enhances dispersion, prevents agglomeration, maintains structural integrity, and reduces structural defects [18,19,70]. In addition, research studies mentioned that DOX release can be initiated at lower pH conditions. Since tumor microenvironments are usually acidic, pH-dependent drug release presents a promising approach for selectively releasing drugs at tumor sites, thus minimizing drug leakage and enhancing therapeutic efficacy [17,71]. Therefore, there is a consistent relationship between our research findings with the previous literature on the degradation and stability of BP under various environmental conditions. This finding supports the potential utility of the synthesized nanocomposite under physiological conditions for biological applications, particularly as an effective therapeutic agent for drug delivery and cancer treatment.

## 3. Materials and Methods

### 3.1. Synthesis of Exf. BP NSs and BP-PEG@Au-GSH-DOX NSs

The nanoscale synthesis of BP-PEG@Au-GSH-DOX was performed according to a previously reported method by our group [31]. BP NSs were synthesized by the simple exfoliation of the corresponding bulk BP. Bulk BP (5.00 mg) was dispersed in 20 mL N-methyl pyrrolidone (NMP) in an Ar glove box. The mixture was sonicated in an ice bath for 5 h using a probe sonicator at 200 W (ultrasonic frequency, 40 kHz). The resulting brown dispersion was centrifuged at 3000 rpm for 15 min to separate unexfoliated BP from the residue. The supernatant was carefully collected and subjected to another round of centrifugation at 6000 rpm for 40 min. Subsequently, the BP NSs precipitate was washed twice with deionized water to remove any remaining NMP. The resuspended BP NSs solution was filtered using a polyester membrane filter (0.2 µm) to obtain uniform-sized BP NSs. The resulting BP NSs were then resuspended in distilled H_2_O. Subsequently, 10 mg of mPEG-NH_2_ was added to exfoliated BP NSs in distilled H_2_O (2 mg in 5 mL). After 30 min of sonication, the mixture was stirred overnight. The resulting reaction mixture was centrifuged at 6000 rpm for 30 min to collect PEG-functionalized BP NSs.

Au(III) chloride (26 mg) was dissolved in 26 mL distilled H_2_O and stirred thoroughly. The gradual addition of glutathione (162 mg) to the solution was carried out under vigorous stirring while cooling the mixture in an ice bath, leading to the formation of a cloudy white Au(III)-based polymer. The mixture turned colorless upon the addition of saturated NaHCO_3_ (3.2 mL). The clear Au-based salt solution was cooled again at 0 °C in an ice bath to rapidly inject NaBH_4_ (50 mg, dissolved in 5 mL ice-cold water) and allowed to react for 2 h until it gradually turned a wine-red color, indicating the reduction of the Au-based salt. Subsequently, MeOH (32 mL) was gradually added with constant stirring to precipitate NPs. The material was collected by centrifugation at 6000 rpm for 40 min. The precipitate was rinsed thrice with methanol (1 mL × 3), followed by a final wash with a mixture of 1 mL MeOH/H_2_O (1:2 *v*/*v*). The resulting material was then dried under reduced pressure in a vacuum desiccator at room temperature.

Au-GSH (50 mg) was dissolved in 10 mL distilled water, and then 4 mg of doxorubicin hydrochloride was added. The mixture was stirred for two nights in an Ar glove box. The resulting mixture was centrifuged at 6000 rpm for 40 min, and the precipitate was collected and washed with phosphate buffer solution until no more doxorubicin hydrochloride was detected by UV-vis absorption spectroscopy. The precipitate was then collected and dispersed in distilled water, H_2_O, for further use.

Then, 2 mL of PEG-modified BP (1 mg/mL) was mixed with 2 mL of Au-GSH-DOX (1 mg/mL), and the pH was adjusted to 8.5 using NaOH. The mixture was stirred vigorously for 2 days in an Ar glove box at room temperature. The resulting product was collected after centrifugation at 6000 rpm for 30 min and washed with distilled water before use.

The surface morphologies of the BP NSs and BP-PEG@Au-GSH-DOX NSs were observed using scanning transmission electron microscopy (STEM, JEM2100F, JEOL, Tokyo, Japan) at 200 kV, while chemical analysis was performed using Raman scattering (NRS-7100, JASCO, Tokyo, Japan) and X-ray photoelectron spectroscopy (XPS, Shimadzu AXIS ULTRA DLD, Kyoto, Japan).

### 3.2. Stability Evaluation of Exf. BP and BP-PEG@Au-GSH-DOX NSs

The degradation stability was evaluated in Exf. BP NSs and BP-PEG@Au-GSH-DOX samples which contained the same BP NSs (20 ppm) in deionized water. The observations and analysis were conducted at three environmental conditions [I. inert conditions in an Ar glove box (23 ± 2 °C, 5 ± 2% RH), II. cooling temperature conditions (4 ± 2 °C, 60 ± 5% RH), and III. ambient atmospheric conditions (23 ± 2 °C, 71 ± 3% RH)] for a period of four weeks. The morphological and chemical properties of the samples were observed at predetermined time intervals using a scanning transmission electron microscope (STEM, JEM2100F, JEOL) at 200 kV, Raman scattering (JASCO, NRS-7100), and X-ray photoelectron spectroscopy (XPS, Shimadzu AXIS ULTRA DLD).

## 4. Conclusions

A detailed evaluation of the morphological and chemical characteristics was conducted to investigate the nanoscale degradation behavior of BP-PEG@Au-GSH-DOX, PEGylated BP NSs, and Exf. BP NSs under three different environmental conditions including an inert environment in an Ar glove box, low-temperature conditions, and ambient conditions over predetermined time intervals. The TEM, XPS, and Raman analyses were employed to investigate the successful functionalization of BP NSs with PEG and Au-GSH-DOX as well as the degradation behavior in synthesized nanoconjugates. The TEM images indicated the presence of BP-PEG@Au-GSH-DOX NSs, approximately 100 nm in size, which were well decorated with Au NPs of around 10 nm in diameter. Raman spectroscopy confirmed successful surface and modification functionalization of BP during the synthesis process. According to the degradation stability evaluation carried out under the three distinct exposure conditions, TEM, XPS, and Raman analysis collectively revealed that the three samples of Exf. BP, PEGylated BP, and BP-PEG@Au-GSH-DOX demonstrated the greatest level of stability in Ar exposure conditions due to controlled exposure to oxidizing agents. Moreover, BP-PEG@Au-GSH-DOX showed enhanced stability compared to Exf. BP and PEGylated BP, owing to the formation of a protective barrier by PEG and Au NPs on the BP surface. This barrier occupied the energetic defect sites and reactive lone pairs, which may have reacted in the presence of oxygen and water molecules. Thus, synthesized BP-PEG@Au-GSH-DOX evidenced the significant utility of the synthesized nanoconjugate with enhanced degradation stability, physiological compatibility, high drug loading capability, and photothermal activity, making it a significant therapeutic agent for cancer treatment in tumor-targeted applications.

## Data Availability

The datasets generated and/or analyzed during the current study are available from the corresponding author upon reasonable request.
